# Sequence diversity of cytotoxic T cell antigens and satellite marker analysis of *Theileria parva* informs the immunization against East Coast fever in Rwanda

**DOI:** 10.1186/s13071-020-04322-9

**Published:** 2020-09-07

**Authors:** David Kalenzi Atuhaire, Walter Muleya, Victor Mbao, Thomas Bazarusanga, Isidore Gafarasi, Jeremy Salt, Boniface Namangala, Antony Jim Musoke

**Affiliations:** 1 Centre for Ticks and Tick-Borne Diseases, Private Bag A130, Lilongwe, Malawi; 2 Department of Biomedical Sciences, School of Veterinary Medicine, University of Zambia, P.O. Box 32379, Lusaka, 10101 Zambia; 3 International Development Research Centre, Eastern and Southern Africa Regional Office, Nairobi, Kenya; 4 Acre Africa, P.O. Box 6782, Kigali, Rwanda; 5 Rwanda Agricultural Board, P.O. Box 5016, Kigali, Rwanda; 6 Global Alliance for Livestock Veterinary Medicines, Doherty Building, Pentlands Science Park, Bush Loan, Penicuik, Edinburgh, EH26 0PZ Scotland, UK; 7 Department of Paraclinical Studies, School of Veterinary Medicine, University of Zambia, P.O. Box 32379, Lusaka, 10101 Zambia; 8 LMK Medical laboratories and consultancies, P.O. Box 33686, Kampala, Uganda

**Keywords:** ECF, Immunization, Muguga cocktail, Sub-structuring, *Theileria parva*, Tp1, Tp2

## Abstract

**Background:**

East Coast fever (ECF) caused by *Theileria parva* is endemic in Rwanda. In this study, the antigenic and genetic diversity of *T. parva* coupled with immunization and field challenge were undertaken to provide evidence for the introduction of ECF immunization in Rwanda.

**Methods:**

Blood collected from cattle in the field was screened for *T. parva* using ELISA and PCR targeting the *p104* gene. *Tp1* and *Tp2* gene sequences were generated from field samples and from Gikongoro and Nyakizu isolates. Furthermore, multilocus genotype data was generated using 5 satellite markers and an immunization challenge trial under field conditions using Muguga cocktail vaccine undertaken.

**Results:**

Out of 120 samples, 44 and 20 were positive on ELISA and PCR, respectively. Antigenic diversity of the *Tp1* and *Tp2* gene sequences revealed an abundance of Muguga, Kiambu and Serengeti epitopes in the samples. A further three clusters were observed on both *Tp1* and *Tp2* phylogenetic trees; two clusters comprising of field samples and vaccine isolates and the third cluster comprising exclusively of Rwanda samples. Both antigens exhibited purifying selection with no positive selection sites. In addition, satellite marker analysis revealed that field samples possessed both shared alleles with Muguga cocktail on all loci and also a higher proportion of unique alleles. The Muguga cocktail (Muguga, Kiambu and Serengeti) genotype compared to other vaccine isolates, was the most represented in the field samples. Further low genetic sub-structuring (F_ST_ = 0.037) coupled with linkage disequilibrium between Muguga cocktail and the field samples was observed. Using the above data to guide a field immunization challenge trial comprising 41 immunized and 40 control animals resulted in 85% seroconversion in the immunized animals and an efficacy of vaccination of 81.7%, implying high protection against ECF.

**Conclusions:**

Antigenic and genetic diversity analysis of *T. parva* facilitated the use of Muguga cocktail vaccine in field conditions. A protection level of 81.7% was achieved, demonstrating the importance of combining molecular tools with field trials to establish the suitability of implementation of immunization campaigns. Based on the information in this study, Muguga cocktail immunization in Rwanda has a potential to produce desirable results.
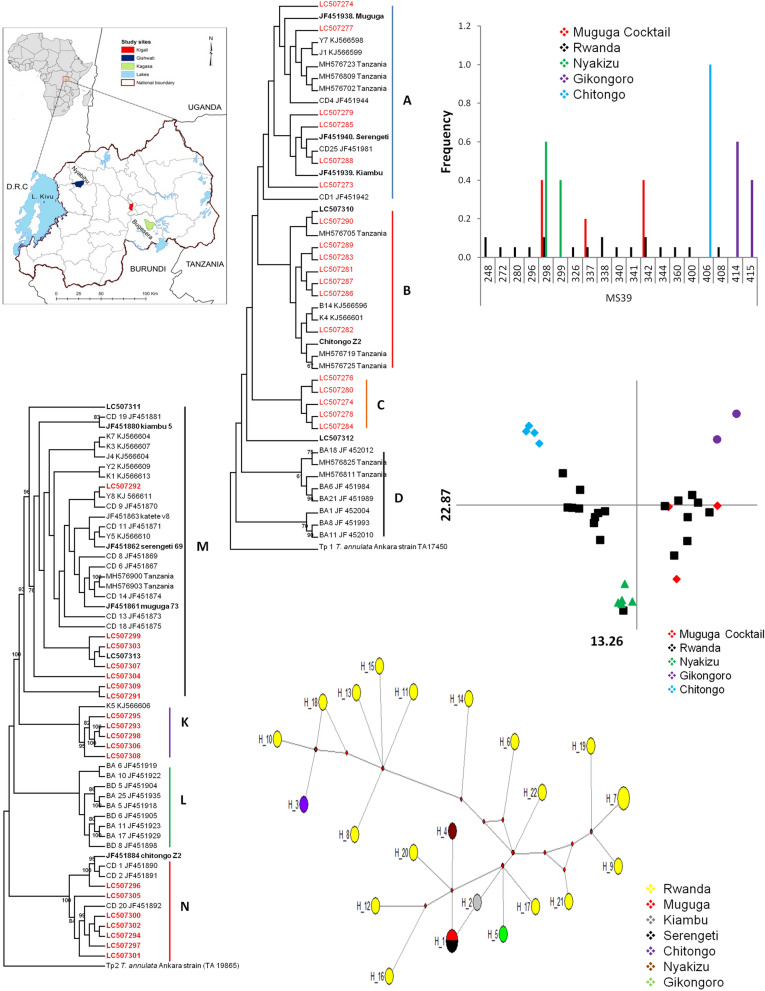

## Background

East Coast fever (ECF) is a disease of cattle caused by *Theileria parva*, a protozoan parasite that is transmitted by the three-host tick *Rhipicephalus appendiculatus* [1]. In eastern, central and southern Africa, ECF is considered to be the most economically important tick-borne disease of cattle [2] accounting for almost half of the deaths in calves in endemic countries and causing losses of up to 1 million cattle and US$300 million in revenue annually [3]. ECF further threatens up to 25 million cattle of which 21 million belong to small scale farmers [3]. The disease is associated with high levels of mortality, especially in improved stocks and indigenous cattle in endemically unstable areas [4], thus it is a major constraint to increasing livestock production through adoption of genetically improved breeds of cattle. In Rwanda, agriculture contributes about 31% to the Gross Domestic Product (GDP), 10% of which is attributed to the livestock subsector [5]. Cattle is considered to be the major livestock species contributing highly to meat production and is the only source of milk [6]. However, breed improvement programmes in Rwanda are hampered by protozoan tick-borne parasites especially ECF [7–9] which has a prevalence of more than 80% [10, 11].

Control of ECF in the country is achieved through a combination of chemotherapy (i.e. buparvaquone) and vector control by use of acaricides on cattle as spray or cattle dips [8]. These control measures are expensive especially in small indigenous low-productivity breeds where the cost may be equivalent to the value of the animal and effective treatment is only possible when the disease is detected early. The stated control measures also tend to promote tick resistance to acaricides and increase susceptibility of cattle to tick-borne diseases [12]. Immunization through the infection and treatment method (ITM), on the other hand, provides lifelong protection in animals and is a cheaper alternative. Unlike other countries in the region, Rwanda has not adopted ITM, although there have been efforts to introduce it using the locally isolated Nyakizu [13] and Gikongoro strains (unpublished observations). With regards to ITM, each region uses a different strain for immunization, for example, Ketete and Chitongo strains are used in the eastern and southern parts of Zambia, respectively, while parts of Tanzania use the Muguga cocktail [14]. Muguga cocktail (MC), a combination of three different *T. parva* isolates (Muguga 73, Kiambu 5 and Serengeti transformed) selected to overcome the strain specificity deficiencies and incomplete cross-protection seen with the use of single parasite isolates [15], is the most widely used and has been shown to reduce calf mortality rates by 90% in Tanzania [16]. Studies have also shown that different epidemiological regions are known to exhibit different parasite population dynamics and degrees of diversity [17–19]. Therefore, determining appropriate live parasite immunization methods must be based on the immunological diversity found in an area. Molecular characterization in this regard provides the best indication of the relatedness of parasites [17, 19].

To date, a number of *T. parva* antigens and epitopes recognized by cytotoxic lymphocytes (CTLs), CD8^+^ T cells, from *T. parva*-immune cattle have been identified [12, 20–22]. Several studies have demonstrated that protection of cattle is mediated by MHC class 1 restricted CTLs that destroy schizont-infected lymphocyte*s* [23, 24]. The most commonly used of these antigens to determine the extent of polymorphism in several isolates of *T. parva* have been *Tp1* and *Tp2* [25–29]. The sequencing of the *T. parva* whole genome has also helped in the identification of a panel of DNA satellite markers that allow more detailed genotyping of the different isolates [18, 30]. Studies on *T. parva* populations from different regions of Uganda, Kenya, Zambia and Tanzania using satellite markers have shown a high degree of diversity and frequency of infection of cattle with mixed genotypes [18, 19, 30] as well minimum or low diversity in the Muguga cocktail vaccine [31]. In as much as these genotypic studies have provided an insight in the genetic structure of *T. parva* populations, they are not informative in relation to the nature and selective pressures driving antigenic diversity relevant to immune protection [25]. However, combining sequence diversity with genetic studies offers a solution for this shortfall and provides more information on the similarities of parasite populations in comparison with vaccine strains. Furthermore, this information also provides a much more accurate guide on how vaccine candidates for field immunization challenge trials can be selected. Therefore, this study aimed to answer the following questions: What is the sequence diversity of *T. parva* *Tp1* and *Tp2* antigens in local breeds of cattle from Bugesera District of Eastern Rwanda? How similar are the *Tp1* and *Tp2* epitopes in field samples from Bugesera District to the epitopes in the Muguga, Kiambu, Serengeti, Chitongo, Gikongoro and Nyakizu vaccine isolates? Does the *T. parva* population from Bugesera District share common alleles with the Muguga, Kiambu and Serengeti (collectively referred to as Muguga cocktail), Chitongo, Gikongoro or Nyakizu vaccine isolates and how closely related are they? and lastly, based on the answers to the above questions, can the chosen vaccine isolate offer a high level of protection in immunized animals exposed to natural field challenge in Bugesera District?

## Methods

### Study sites

**Fig. 1:**
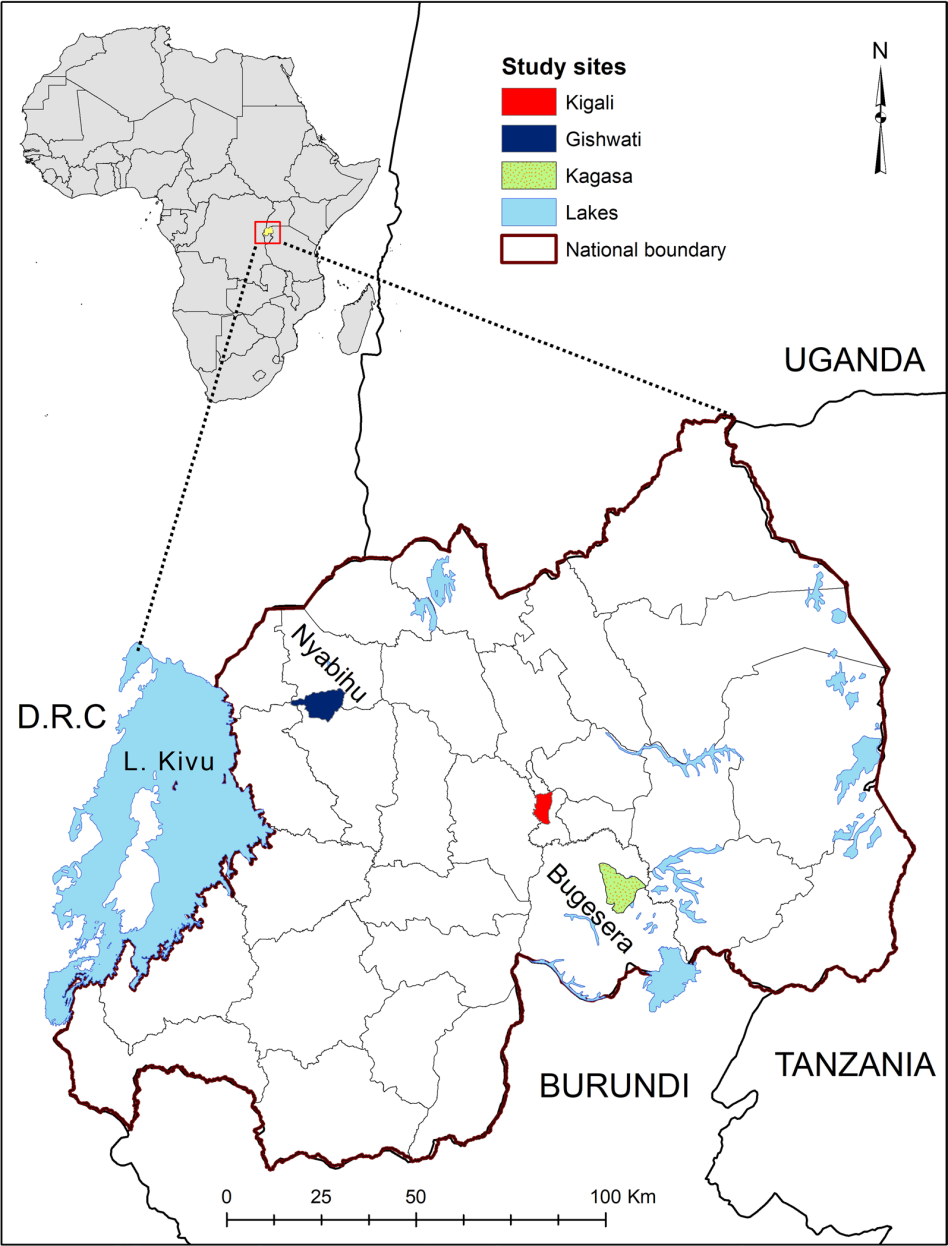
Map of Rwanda showing the location of study sites

Rwanda, an east African country located between latitudes 1°04′ and 2°51′ South and longitudes 28°45′ and 31°15′ East, comprises the following administrative provinces: Eastern; Western; Southern; Northern; and Kigali city. Field samples were collected from local breeds of cattle from Bugesera District in the eastern part of the country (Fig. 1). Cattle for the immunization and challenge study were sourced from the highland area of Gishwati in Nyabihu District and then transported to the Rwanda Agricultural Board (RAB) quarantine facility in Kigali for immunization. After immunization, the animals were then relocated to Karama farm in Kagasa village, Bugesera District, in the Eastern province of Rwanda.

### Cattle blood sampling and handling

Paired blood samples were collected in plain and EDTA tubes from 128 animals for serology and PCR analysis, respectively. Sera were extracted from plain tube blood samples by centrifugation at 2500× *rpm* for 10 min and stored at − 20 °C. EDTA-treated blood drops were spotted onto Whatman filter paper (Sigma-Aldrich^®^, St. Louis, Missouri, USA) and air-dried in the shade overnight. The Filter papers were then stored in Ziploc^®^ plastic bags filled with silica gel and sent to the Centre for Ticks and Tick-Borne Diseases in Lilongwe, Malawi where subsequent genomic DNA extraction, using a commercial kit (Thermo Fisher Scientific^®^, Waltham, Massachusetts, USA) as well as PCR analysis was carried out [32, 33]. An aliquot of *T. parva* positive DNA was then sent to the University of Zambia, School of Veterinary Medicine where further analysis was carried out.

### ELISA screening of *Theileria parva* and other tick-borne haemoparasites

Antibodies to *T. parva* were detected in sera using a recombinant PIM-based ELISA kit as previously described [34]. The ELISA results were read as optical densities (OD) obtained from the analysis of the samples in a Titertek Multiskan Mcc340 spectrophotometer (Thermo Fisher Scientific^®^, Waltham, Massachusetts, USA). Controls were included in every test. Antibodies to other tick-borne haemoparasites (*Theileria mutans, Babesia bovis, Babesia bigemina* and *Anaplasma marginale*) were also detected using the same procedure previously described for *T. parva* [34] except that the respective recombinant antigenes were used.

### PCR screening of *Theileria parva*

The presence of *T. parva* genomic DNA in field samples was assessed using PCR targeting the *p104 *gene. PCR was carried out using the Amplitaq Gold master mix PCR kit (Invitrogen^®^, Waltham, Massachusetts, USA) according to the manufacturer’s instructions and the *T. parva* specific *p104* gene primers earlier described [35]. The PCR cycles were; 10 min denaturation at 95 °C followed by 35 cycles of 96 °C for 60 s, 63 °C for 30 s and 68 °C for 60 s, with a final extension step at 72 °C for 5 min. The amplified products were analyzed on agarose gel (1.5%) coated with ethidium bromide.

### PCR amplification of *Tp1* and *Tp2* genes

*Theileria parva Tp1* and *Tp2* genes were amplified from p104 positive field samples using Amplitaq Gold master mix PCR kit (Invitrogen^®^, Waltham, Massachusetts, USA). A 20 µl reaction mix prescribed by the manufacturer was used and the primers utilized as described [25]. The PCR cycles included; initial denaturation at 95 °C for 10 min followed by 40 cycles at 96 °C for 30 s, 50 °C (*Tp1*) and 53 °C (*Tp2*) for 45 s and 68 °C for 60 s with an extension step of 5 min at 72 °C. Agarose gel coated with ethidium bromide was then used to visualize amplified PCR products.

### Cycle sequencing

Cycle sequencing was achieved using the BigDye Terminator v3.1 cycle sequencing kit (Life Technologies^®^, Carlsbad, California, USA). Prior to this, excess buffers and dNTPs were purified from the PCR products using the Monofas purification kit (GL Sciences, Tokyo, Japan) according to the manufacturer’s instructions. After successful cycle sequencing PCR, any excess labeled dNTPs as well as buffers were removed from the sequence products using the ethanol precipitation method. The resultant purified sequence products were then subjected to capillary electrophoresis using the ABI 3500 genetic analyzer (Applied Biosystems^®^, Waltham, Massachusetts, USA).

### Microsatellite PCR

The markers used in genotyping field samples as well as the Muguga, Kiambu, Serengeti, Chitongo, Gikongoro and Nyakizu vaccine isolates are listed in Table 1. All the forward primer markers were fluorescently labeled with ABI compatible dyes. Amplitaq Gold master mix PCR kit (Invitrogen^®^, Waltham, Massachusetts, USA) was used to amplify the repeat regions of the *T. parva* genome in a 20 µl reaction according to the manufacturer’s instructions. The annealing temperatures used for each marker are as described previously [36]. The PCR conditions used were denaturation at 95 °C for 10 min followed by 35 cycles of 96 °C for 1 min, annealing for 30 s and 68 °C for 1 min with a final extension of 72 °C for 5 min. Successful PCR products were analyzed on 1.5% agarose gel pre-stained with ethidium bromide. Amplified PCR products were denatured and then electrophoresed on the ABI Seqstudio genetic analyzer (Applied Biosystems^®^, Waltham, Massachusetts, USA). Fragment DNA sizes from the field samples were analyzed using the GeneMapper software ver. 5 (Applied Biosystem, Waltham, Massachusetts, USA). The allele with the highest area under its peak was taken as the most dominant allele and was used to construct a multi-locus genotype (MLG) representing the most dominant genotype within each sample.

**Table 1:** Satellite markers used to genotype field samples from Rwanda, Muguga, Kiambu, Serengeti, Gikongoro and Nyakizu vaccine stocks

Marker	Chromosome number	Size (bp)
ms9	3	230
MS7	1	372
MS19	2	307
MS25	3	325
MS39	4	263

*Note*: The satellite markers used in this study were described by Patel et al. [36]

### Immunization-challenge field trial

#### Animals

Eighty-nine Friesian calves (*Bos Taurus*) aged 6 to 8 months old and free from antibodies to *T. parva* [27] and negative on p104 PCR were acquired from the highland area of Gishwati (North-West) where no history of ECF is recorded. The calves were quarantined at the Rwanda Agriculture Board (RAB) facility in Kigali for a 4-week period. During this period, the animals were kept under intensive acaricide control for ticks and fed on fumigated hay, commercial feed concentrate, mineral supplements and water *ad libitum*. Prior to immunization, the calves were dewormed using a combination of albendazole and ivermectin. An active surveillance of possible ECF infection was carried out through daily rectal temperature records, weekly serology and parasitological analysis, routine inspection of ticks and clinical examination.

#### Vaccine stabilate

The Muguga cocktail vaccine, designated as MCL01, used for this immunization challenge trial was produced as the first commercial batch of vaccine at the Centre for Ticks and Tick-Borne Diseases (CTTBD) in Lilongwe, Malawi in 2013 following technology transfer from International Livestock Research Institute (ILRI), Nairobi, Kenya. The vaccine was transported in liquid nitrogen to the RAB laboratory in Rwanda for this purpose.

#### Viability testing of the vaccine

Two calves testing negative to *T. parva* antibodies were used to validate the viability of the vaccine. Each animal received 1 ml of a 1:100 diluted dose of MCL01 vaccine subcutaneously below and in front of the parotid lymph gland. The reaction to ECF live vaccine injection was recorded as previously described [37]. In short, the reaction to ECF infection was assessed daily by clinical inspection and parasitological examination. Clinical inspection involved the assessment of general body condition, swollen lymph nodes and presence of pyrexia coupled with other clinical signs such as the loss of appetite and change in respiration rate. Parasitological examination involved the detection of schizonts and piroplasms from lymph gland biopsy and blood smears, respectively.

#### Inoculation of calves

Calves (*n* = 81) were ear tagged, weighed and randomly allocated into the control (*n* = 40) and immunized (*n* = 41) groups. The immunization procedure was carried out using the method previously described [38]. The immunized group was injected with 1 ml of a 100× dilution of MCL01 stabilate. The vaccine was inoculated subcutaneously in front of the right parotid lymph node and treated simultaneously with 30% long acting oxytetracycline (Tetroxy L.A, Bimeda, Dublin, Ireland) at a dosage rate of 30 mg/kg body weight by deep intramuscular injection. The control group was injected with an equivalent dose of the vaccine diluent. Tick control using acaricide spray was applied till day 32 post-immunization.

#### Monitoring of animals

Rectal temperatures of calves were recorded daily after challenge. On day 5 after inoculation, and at daily intervals thereafter, needle biopsy smears were made from the parotid lymph node next to the site of injection. These were then stained with Giemsa and examined for the presence of schizonts under a microscope. The parasite burden was detected in 20 microscopic fields and scored on a scale from 1 to 10. Biopsy smears were similarly taken daily from the contralateral pre-scapular lymph node from the day after the draining lymph node was found positive with schizonts. Blood collected from the jugular vein was used to prepare blood smears and serum samples. Blood smears were prepared daily from the day after an animal first became positive with schizonts. These smears were stained with Giemsa and piroplasm parasitaemia was determined as the number of infected erythrocytes per 1000 cells. Weekly ELISA examination of sera was also carried out to measure the antibody response to ECF infection [34].

#### Exposure of animals to field challenge

On the 21st day post-immunization, animals were relocated to Karama field station, another RAB enclosed facility in the Eastern Province for field exposure. The same monitoring protocol of ECF reaction was applied during the 2 weeks acclimatization period. Prior to exposure of animals to field tick challenge, calves were thoroughly washed for 3 consecutive days with water and soap to remove any excess acaricide. On day 35 post-immunization, animals were allowed to freely graze at Karama farm, a RAB facility known to be highly infested with *T. parva* infected ticks [39]. The success in tick attachment was assessed by conducting a quick inspection of animals at the time of taking daily temperature readings in the first week following the commencement of field exposure. Based on the ECF reaction index previously described [37], severely reacting animals were humanely euthanized. *Post-mortem* examination was carried out on each animal to discriminate between ECF and non ECF causes. Animals less severely affected were allowed to recover.

#### Determination of vaccine safety and efficacy of vaccination

Blood was collected on day 0, prior to vaccination and on day 35 post-vaccination for the purpose of estimating antibody titres in both the vaccinated and control groups. The levels of seroconversion for the two groups as well as the ECF reactors between day 1 and day 35 were recorded. The field exposure period lasted 90 days, at the end of which each animal was categorized as non-reactor (NR), mild reactor (MR), moderate reactor (MODR) or severe reactor (SR) based on an overall assessment of the level of pyrexia, parasitosis and postmortem findings. The fraction of severe reactors in the vaccinated and control groups were then used to calculate the vaccine efficacy using a formula described previously [40].

$$\% {\text{ Efficacy }} = \frac{{\left( {{\text{Fraction of severe reactors in control }}{-}{\text{ Fraction of severe reactors in vaccinated}}} \right)}}{\text{Fraction of severe reactors in control}} \times { 1}00\%$$

### Data analysis

#### ELISA

The OD values were expressed as percent positivity (PP) relative to a strong-positive control serum reference [41]. Any test serum with a PP value of 20 or above was considered positive as previously described [34].

#### Sequence analysis

Nucleotide sequences obtained from field samples as well as from the Gikongoro and Nyakizu vaccine isolates were first subjected to NCBI BLAST analysis, then edited and finally assembled using the ATGC software plug-in in Genetyx ver. 12 (Genetyx Co., Tokyo, Japan). Furthermore, Muguga, Kiambu, Serengeti and other reference sequences were downloaded from the GenBank and utilized together with the nucleotide sequences obtained in this study to generate multiple sequence alignments of *Tp1* and *Tp2* using Clustal W1.6. From the nucleotide sequences obtained, amino acid sequences were translated and *Tp1* and *Tp2* multiple amino acid sequence alignment files generated for the purpose of determining the similarity of the vaccine epitopes *versus* those expressed by field samples. A fasta file of the multiple sequence alignments for *Tp1* and *Tp2* genes was further converted to a mega file and utilized to generate a neighbor joining phylogenetic tree for each gene with a confidence level of 1000 bootstrap replicates using MEGA 6 computer software [42].

The average number of nucleotide differences per site for each gene was calculated using DnaSP ver. 5 [43] and the type of selection pressure occurring on both genes was determined by the ratio of the non-synonymous substitutions and synonymous substitution (dN/dS) per site using the single likelihood ancestor counting (SLAC) method with a 0.05 confidence level and F81model. The output data was interpreted as dN/dS = 1 for neutrality, < 1 for negative selection and > 1 for positive selection. The data monkey interface website was used to run all analyses (http://www.datamonkey.org) [44]. Nucleotide sequences from this study were deposited in the DNA Data Bank of Japan (DDBJ) with accession numbers LC507273-LC507313 (Additional file 1: Table S1).

#### Microsatellite analysis

Muguga, Kiambu and Serengeti vaccine isolates were each subjected to satellite PCR using the five markers listed in Table 1 followed by capillary electrophoresis and the data obtained from each isolate was combined so as to form a single population referred to as Muguga cocktail (MC). Microsatellite toolkit (http://animalgenomics.ucd.i.e./sdepark/ms-toolkit/) was used to perform initial similarity analysis of the multi-locus genotype (MLG). A principal components analysis (PCA) to visualize the similarities among the field samples and the Muguga, Kiambu and Serengeti (collectively referred to as Muguga cocktail) as well as Chitongo, Gikongoro and Nyakizu vaccine isolates was constructed using GenAIEx6 [45]. Furthermore, the level of sub structuring among field samples, MC, Chitongo, Gikongoro and Nyakizu was determined by calculating estimates for population genetic analysis (F statistics) using the FSTAT computer package version 2.9.3.2 (https://www2.unil.ch/popgen/softwares/fstat.htm). The null hypothesis of linkage equilibrium (LE) and panmixia (random mating) was assessed using LIAN (http://adenine.biz.fh-weihenstephan.de/lian/). LIAN calculates the standardized index of association (ISA) [46] as well as the variance of pairwise differences (V_D_) between data and the variance of differences expected for panmixia (V_E_) and L (the 95% confidence interval for V_D_). The index of association is a measure of association between alleles at loci pairs and values closer to zero or negative indicate panmixia while values significantly greater than zero indicate non-panmixia. Comparison of the V_D_ value *versus* the L value guides whether the null hypothesis of panmixia is accepted or rejected. When the calculated V_D_ value is greater than the L value, the null hypothesis of panmixia is discarded and linkage disequilibrium (LD) is indicated, and when it is less than the calculated L value, panmixia and linkage equilibrium (LE) is indicated.

## Results

### Serology and PCR results

Out of the 128 cattle sampled 44 (34.4%) were positive for *T. parva* antibodies, 45 (35.2%) were positive for *T. mutans*, 14 (11%) were positive for *Babesia bigemina*, 7 (8.6%) were positive for *B. bovis* and 27 (21.1%) were positive for *Anaplasma marginale.* Only 20 samples were positive on *p104* *T. parva* PCR (Table 2).

**Table 2:** ELISA detection of tick-borne haemoparasites and molecular detection of *T. parva* *p104* gene by PCR in blood samples from 128 cattle from Eastern Rwanda

Assay	Tick borne haemoparasites screened (*n* = 128)				
	*Theileria parva*	*Theileria mutans*	*Babesia bigemina*	*Babesia bovis*	*Anaplasma marginale*
ELISA	44 (34.4%)	45 (35.2%)	14 (11%)	7 (8.6%)	27 (21.1%)
p104 PCR	20 (15.6%)	na	na	na	na

*Abbreviation*: na, not applicable

### *Tp1* locus

The complete 432bp *Tp1* gene of *T. parva* was successfully sequenced from 18 out of 20 *p104* gene-positive samples. The obtained nucleotide sequences were translated into amino acid sequences which were then used to compare with Muguga, Kiambu, Serengeti, Chitongo, Gikongoro and Nyakizu amino acid sequences. The epitope VGYPKVKEEML present in Muguga, Kiambu and Serengeti vaccine isolates [25] and VGYPKVKEEII present in Chitongo were identified in 11 and 6 samples, respectively (Additional file 2: Figure S1). Nyakizu also shared 100% epitope homology with Muguga, Kiambu and Serengeti vaccine isolates. On the other hand, Gikongoro and sample RW19 both possessed the epitope VGYPKVKEEMI that was previously reported in Kenya [25]. No other epitopes were identified thus the majority of the samples (11/18) shared 100% epitope sequence homology with the components of Muguga cocktail namely Muguga, Kiambu and Serengeti.

**Fig. 2:**
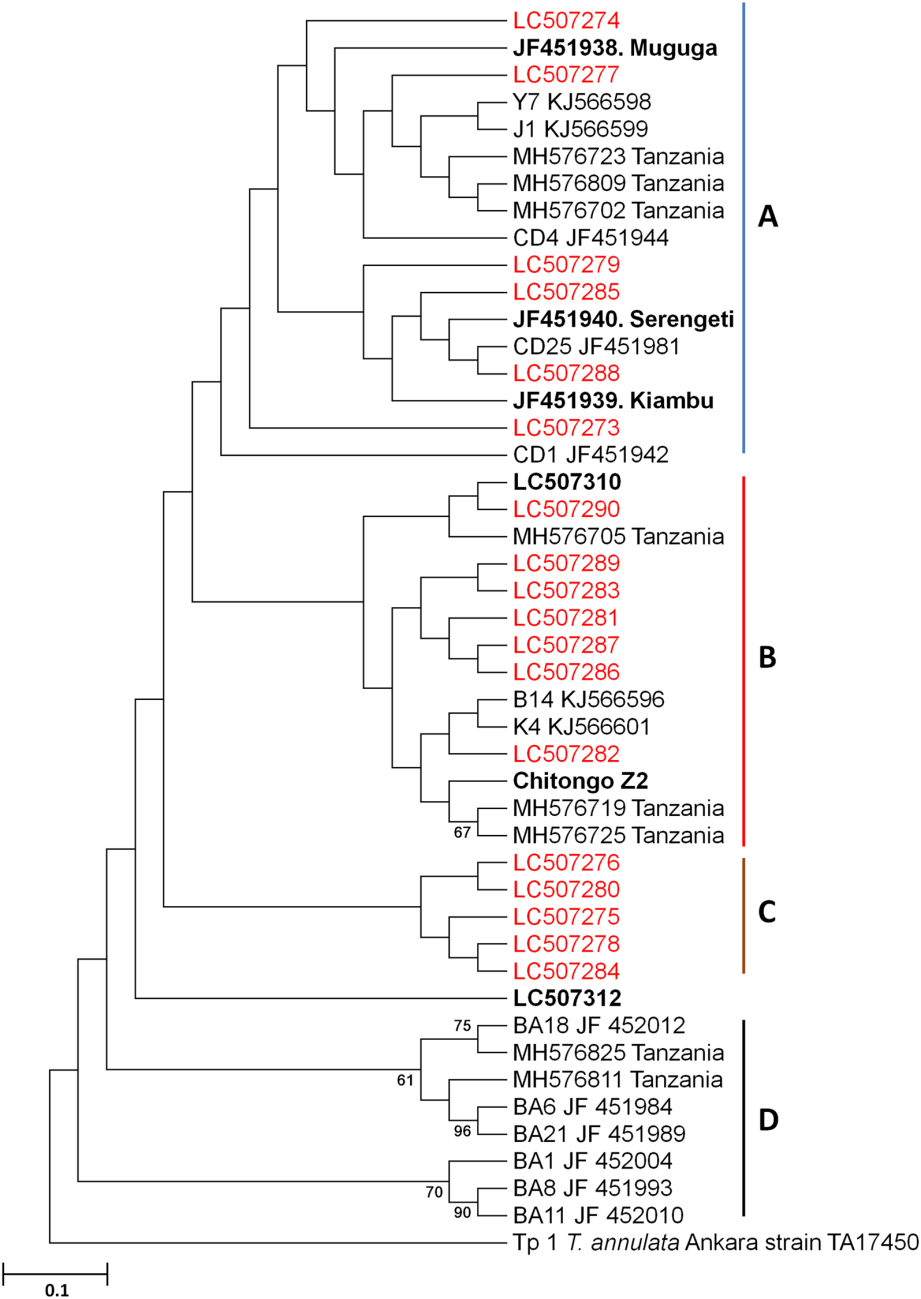
*Theileria parva Tp1* neighbor-joining phylogenetic tree generated from 432 bp nucleotide sequences using MEGA 6 with 1000 bootstrap replicates. Rwanda *Tp1* sequences are given in red and Muguga, Kiambu, Serengeti, Chitongo, Gikongoro and Nyakizu vaccine isolates are given in black and bold. Bootstrap values greater than 65% are shown on the phylogram. The outgroup used on the phylogenetic tree is *Tp1 Theileria annulata* (GenBank: TA17450)

Phylogenetic analysis based on the *Tp1* nucleotide sequences showed 4 clusters namely A, B, C and D (Fig. 2). In cluster A, field samples (*n* = 6) clustered with Muguga, Kiambu and Serengeti vaccine isolates as well as with reference sequences from Kenya, Tanzania and South Sudan. In cluster B, field samples (*n* = 7) together with the Gikongoro isolate (Accession number LC507310, Fig. 2) formed a cluster with Chitongo and sequences from Tanzania and South Sudan (Fig. 2). Cluster C was independent of cluster A and B and only consisted of field samples (*n* = 5). The Nyakizu isolate (accession number LC507312, Fig. 2) did not cluster with sequences in A, B or C, however it was closely related to these clusters. None of the samples under study clustered with buffalo derived or associated strains in cluster D (Fig. 2). A DNA polymorphism and dN/dS mean ratio of π = 0.9% and 0.78 with 3 negative selection sites, respectively was observed. There were no positive selection sites detected.

### *Tp2* locus

The complete *Tp2* gene (531 bp) of *T. parva* was successfully sequenced from 19 *p104* gene-positive field samples. This gene encodes the 174 amino acid protein of the Muguga reference sequence (GenBank: XP_765583) with 6 identified epitopes namely CTL 1 (SHEELKKLGML), CTL 2 (DGFDRDALF), CTL 3 (KSSHGMGKVGK), CTL 4 (FAQSLVCVL), CTL 5 (QSLVCVLMK) and CTL 6 (KTSIPNPCKW) [40, 41]. On epitopes 1, 2, 3, 4, 5 and 6, the total number of samples that possessed 100% similarity with epitopes present in Muguga, Kiambu and Serengeti was 5, 5, 5, 3, 3 and 6, respectively (Additional file 2: Figure S2). With regards to the Chitongo vaccine isolate, only sample RW6 (Additional file 2: Figure S2) had 100% sequence homology with Chitongo on all 6 epitopes. The remaining samples only had similar epitope sequences with Chitongo on one or more epitopes but not on all 6 epitopes. Overall, the number of samples that had 100% sequence homology with Chitongo on epitopes 1, 2, 3, 4, 5 and 6 were 1, 7, 1, 1, 1 and 7, respectively (Additional file 2: Figure S2). The Gikongoro epitope 1 (SDEELDKLGML) and 2 (DGFDRNALF) sequences were different from those on Muguga, Kiambu and Serengeti but on the remaining 3, 4, 5 and 6 epitopes, it shared 100% sequence homology with Muguga, Kiambu and Serengeti. Furthermore, across the 6 epitopes of *Tp2*, Gikongoro vaccine isolate did not share 100% homology with Chitongo. On the other hand, the Nyakizu vaccine isolate shared 100% sequence homology with Muguga, Kiambu and Serengeti across all epitopes (Additional file 2: Figure S2). Other epitopes that were different from Muguga, Kiambu, Serengeti and Chitongo were also identified (Table 3). Compared to Chitongo, the epitopes that were similar to Muguga, Kiambu and Serengeti were more represented in the field samples even though the majority of epitopes identified in the field samples were different from those present in Muguga, Kiambu, Serengeti and Chitongo (Table 3). With the exception of SDNELDTLGLL (epitope 1), KSSHGMGKIGR (epitope 3), LAASIKCVS (epitope 4) and ASIKCVSHH (epitope 5) and EGFDKEKLF (epitope 2) and FAQSIMCVL (epitope 4) which were previously reported in Kenya and South Sudan [12, 22] respectively, these epitopes have not been reported elsewhere.

**Table 3:** *Tp1* and* Tp2* epitopes detected from Rwanda field samples in this study

Gene	Epitope variant					
Tp1	**CTL 1**					
	VGYPKVKEEM**I**					
Tp2	**CTL 1**	**CTL 2**	**CTL 3**	**CTL 4**	**CTL 5**	**CTL 6**
	S**DD**ELKKLGM**V (RW18)**	DGFDR**NT**LF **(RW 19)**	KSSHGMGK**I**G**R (RW 18)**	FAQS**I**VCVL **(RW 9)**	QSLVCVL**V**K **(RW 13)**	K**PD**IPNPCKW **(RW 18)**
	**TE**EELKK**M**GM**V (RW3, 8)**	DG**S**DR**N**TLF **(RW 1)**	**I**SSHGMGKVGK **(RW 19)**	FAQSL**M**CV**I (RW 5, 18)**	QS**I**VCVLMK **(RW 9)**	**I**T**D**IPNPCKW **(RW 5)**
	S**D**EEL**GY**LGM**V (RW 5)**	**E**GFD**K**D**T**LF **(RW 5, 18)**	**L**SSHGMGKVGK **(RW 1)**	FAQSL**M**CV**S (RW 19)**	QSL**M**CV**S**M**Q (RW 19)**	**VND**IPNPCKW **(RW 3, 8)**
	**TED**ELKK**M**GM**V (RW 16)**	**E**GFD**KEK**LF **(RW 3, 16, 8)**	KSS**KS**MG**I**VG**R (RW 3, 8)**	F**V**QS**IM**CV**I (RW 3, 8)**	QSL**M**CV**IH**K **(RW 18)**	**V**T**Y**IPNPCKW **(RW 16)**
	SHEEL**DT**LGML **(RW 1)**		KSS**QS**MG**I**VG**R (RW 5, 16)**	F**V**QSL**M**CV**I (RW 16)**	QSL**M**CV**IN**K **(RW 5, 16)**	KTS**V**PNPC**D**W **(RW 1)**
	S**D**EEL**N**KLGM**V (RW 19)**		LTSHGMG**K**IGR **(RW 4, 7, 10, 11, 12, 15)**	FAQS**IM**CVL **(RW 1)**	QS**IM**CV**IN**K **(RW 3, 8)**	
	SDDELDTLG**L**L **(RW 11)**			**L**A**A**S**IK**CV**S (RW 4, 10, 12, 7, 15)**	QS**IM**CVLMK **(RW 1)**	
	SD**N**ELDTLG**L**L **(RW 4, 7, 10, 12)**			**L**A**A**S**IM**CV**S (RW 11)**	ASIKCV**SHH (RW 4, 10, 12, 7, 15)**	
	S**DN**EL**DT**LGML (**RW15**)				ASI**M**CV**SHH (RW 11)**	

*Notes*: Polymorphic amino acids are given in bold and the sample ID for each sample is given in parentheses

**Fig. 3:**
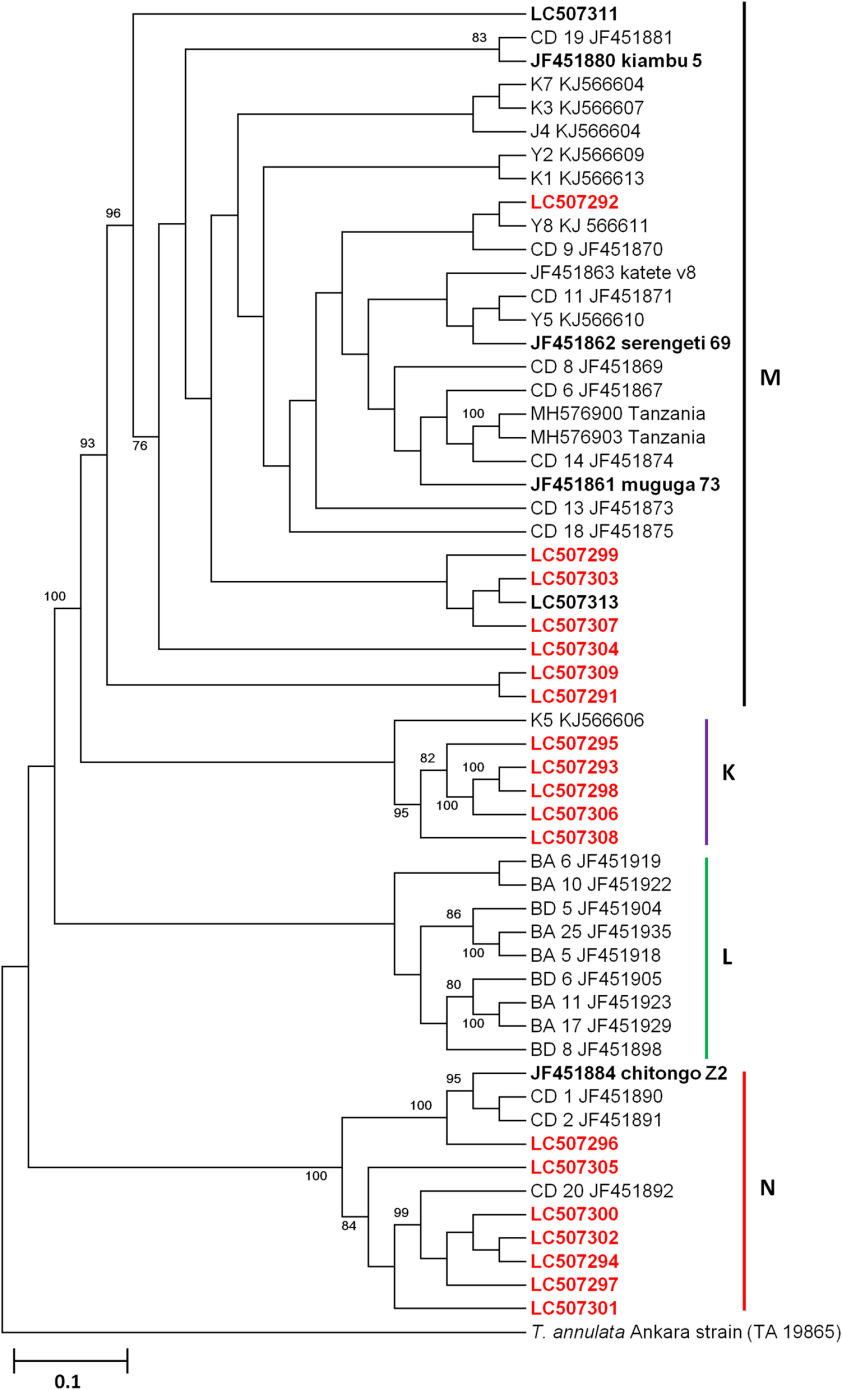
*Theileria parva Tp2* neighbor-joining phylogenetic tree generated from 531 bp nucleotide sequences. MEGA 6 with 1000 bootstrap replicates was used to generate the phylogenetic tree. The sequences from Rwanda are given in red and the Muguga, Kiambu, Serengeti, Chitongo, Gikongoro and Nyakizu isolates in black and bold. Bootstrap values above 65% are given on the phylogram. *Theileria annulata* (GeneBank: TA19865) was utilized as the outgroup

Phylogenetic analysis of the *Tp2* gene showed 4 clusters, namely M, K, L and N (Fig. 3). Cluster M comprised of field samples (*n* = 7), Muguga, Kiambu, Serengeti, Nyakizu, Gikongoro and reference sequences from Kenya, South Sudan and Tanzania, with field samples with accession numbers LC507299, LC507303 and LC507307 closely clustering with the Nyakizu isolate (accession number LC507313) (Fig. 3). Cluster K consisted mainly of field samples (*n* = 5) closely clustering with the reference sequence KJ566609 from South Sudan. The remaining field samples (*n* = 7) formed cluster N with Chitongo and JF451892 from Kenya. Within this cluster, sample LC507296 was closely associated with Chitongo while the remaining samples formed a minor cluster with JF451892 (Fig. 3). All the field samples under study were absent from cluster L which was made up of buffalo derived or associated reference sequences. Further, the DNA polymorphism calculation of π = 15.9% and a ratio of dN/dS = 0.597 with 11 negative selection sites and no positive selection sites was observed.

### Haplotype similarity

**Fig. 4:**
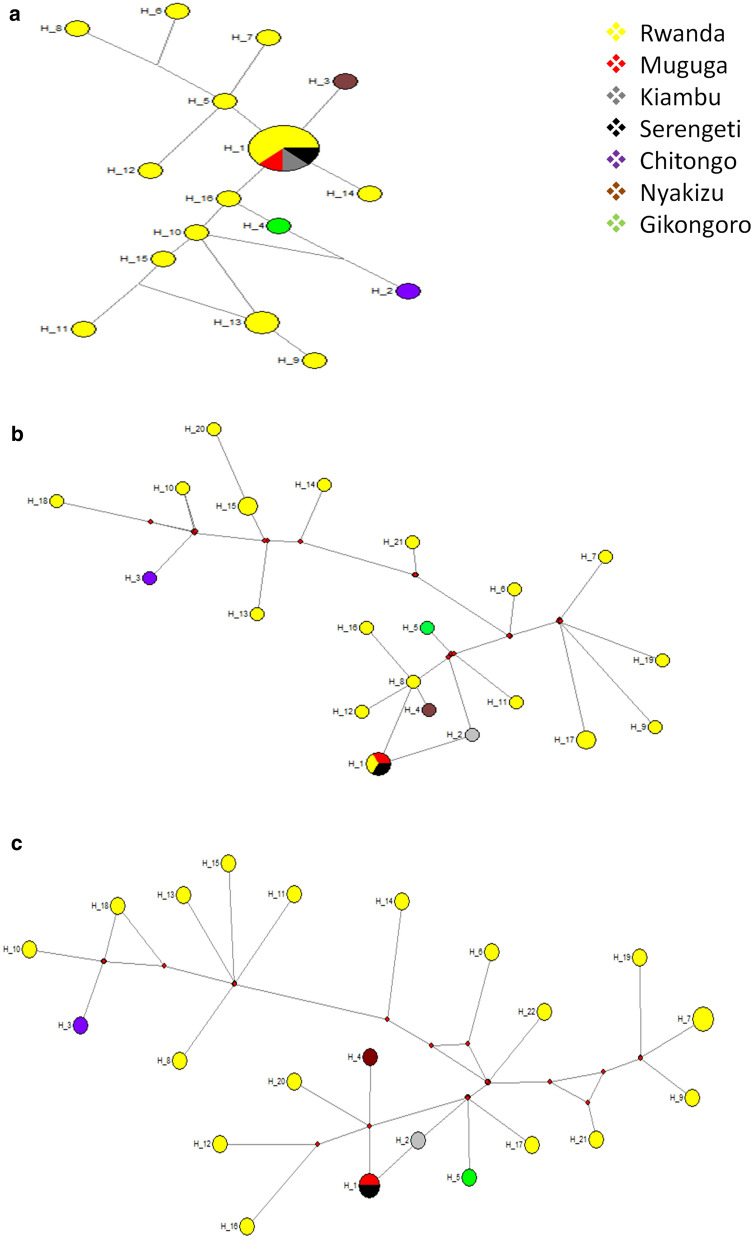
Median-joining network of *Tp1* (**a**), *Tp2* (**b**) and concatenated *Tp1* and *Tp2* sequences from Rwanda and Muguga cocktail, Chitongo, Gikongoro and Nyakizu isolates (**c**). The network compares the relatedness of the Rwanda field samples and the vaccine isolates. The frequency of the haplotype is represented by the size of the circle. Rwanda samples are given in yellow, Muguga cocktail in red, Chitongo in purple, Gikongoro in green and Nyakizu in brown. The size of the circles is proportional to the haplotype frequency

The haplotypes present in Muguga, Kiambu and Serengeti vaccine isolates (collectively referred to as the Muguga cocktail) as well as those in Chitongo, Gikongoro and Nyakizu together with the field samples were analyzed via a median joining (MJ) network constructed using Network ver. 5 (Fig. 4a). Haplotype H1 comprised of Muguga, Kiambu and Serengeti as well as field samples (*n* = 5) and represented the anchor of the network. Haplotypes H2, H3 and H4 comprised of Chitongo, Nyakizu and Gikongoro, respectively while haplotypes H5 to H16 exclusively comprised of field samples. Haplotypes H3, H5, H14 and H16 were observed to radiate from H1 indicating a close and direct relationship to H1. However, H4 was only linked to H1 through H16, and H2 to H1 *via* H4 and H16 (Fig. 4a). Apart from the 5 field samples that shared haplotype H1 with Muguga, Kiambu and Serengeti vaccine isolates, none of the other field samples shared similar haplotypes with Muguga, Kiambu, Serengeti, Chitongo, Nyakizu or Gikongoro vaccine isolates. The most represented haplotype was thus H1 comprising of 8 sequences followed by H13 with 2 sequences. The radiating pattern observed indicated the presence of an expanding population (Fig. 4a).

With regards to *Tp2*, the MJ network (Fig. 4b) constructed using Network ver. 5 was similarly utilized in the analysis of the haplotypes in the field samples as well as in Muguga, Kiambu, Serengeti, Chitongo, Gikongoro and Nyakizu vaccine isolates. Haplotype H1 comprised of Muguga, Serengeti and 1 field sample (*n* = 1) while haplotypes H2, H3, H4 and H5 comprised of Kiambu, Chitongo, Nyakizu and Gikongoro vaccine isolates, respectively. The remaining haplotypes H6 to H21 exclusively comprised of field samples with H15 and H17 comprising of 2 samples each. H1 formed the anchor of the network and only H2 and H8 were directly linked to H1 (Fig. 4b). Haplotype H8 had direct linkages to H4, H12 and H16 and further acted as an indirect link for H4, H12 and H16 to H1. In the same manner, the remaining haplotypes were indirectly linked to H1 *via* H2 and H8 through a series of median vectors (Fig. 4b). The haplotype with the highest frequency was H1, followed by H15 and H17. Only 1 field sample shared the H1 haplotype with Muguga and Serengeti. The remaining samples did not share any direct relationship with H1 and as such were not closely related to H1. Further, a MJ network generated from concatenated *Tp1* and *Tp2* sequence data (Fig. 4c), showed that the haplotypes in field samples were indirectly linked to the vaccine haplotypes H1, H2, H3, H4 and H5 *via* median vectors thus implying a distant relationship among these haploytpes (Fig. 4c).

### Marker diversity, allelic variation and similarity analysis between Muguga cocktail and field samples

**Fig. 5:**
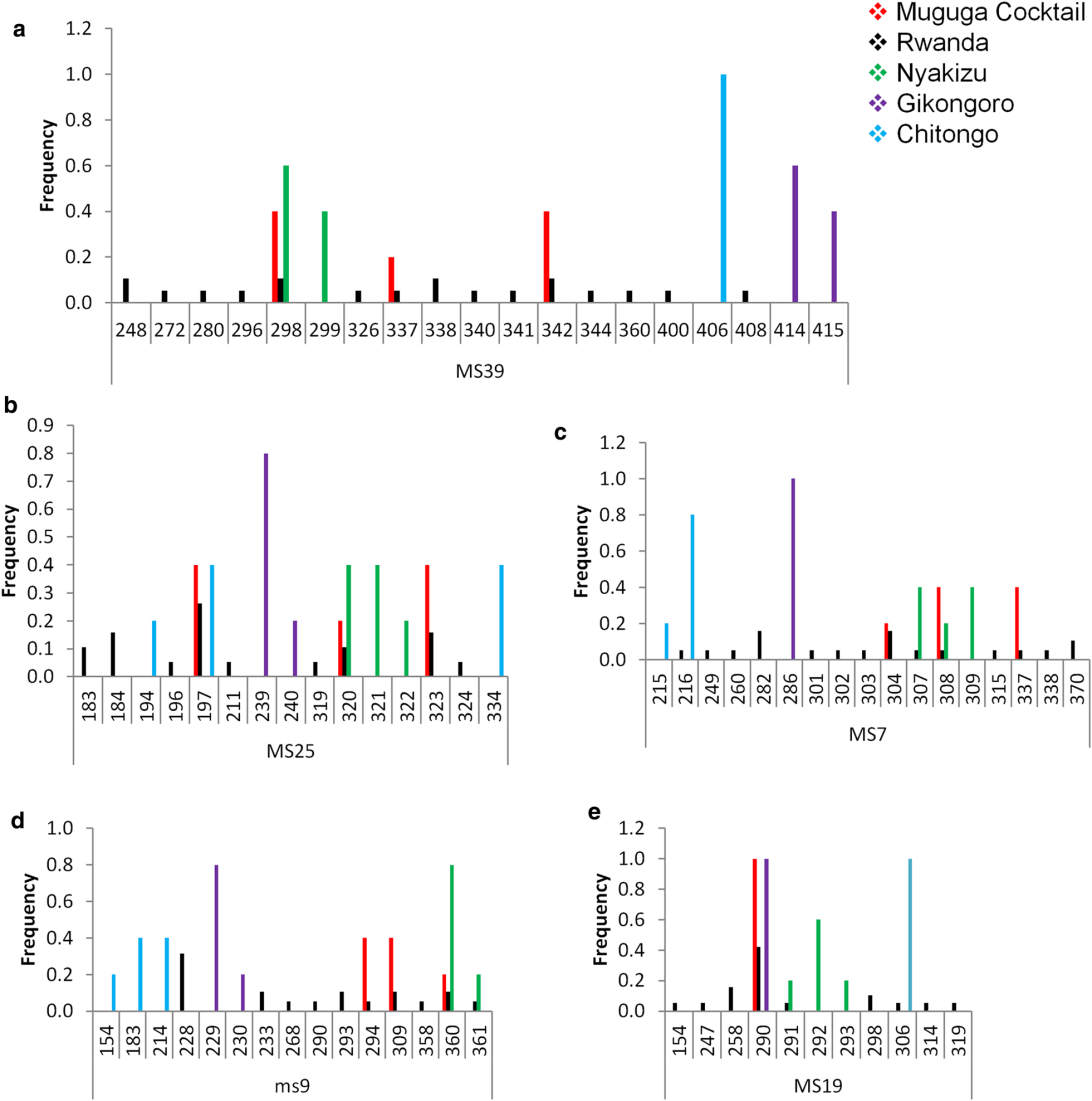
Allele frequencies from field samples, Muguga, Kiambu and Serengeti (collectively referred to as Muguga cocktail), Chitongo, Gikongoro and Nyakizu vaccine isolates. Allele frequencies for loci MS39, MS25, MS7, ms9 and MS19 are presented in **(a**–**e)**, respectively. On each locus, a high proportion of unique alleles is observed with only a few shared alleles. Muguga cocktail and Nyakizu isolates share alleles with Rwanda field samples on all loci while Gikongoro and Chitongo do not (**a**–**e**)

**Fig. 6:**
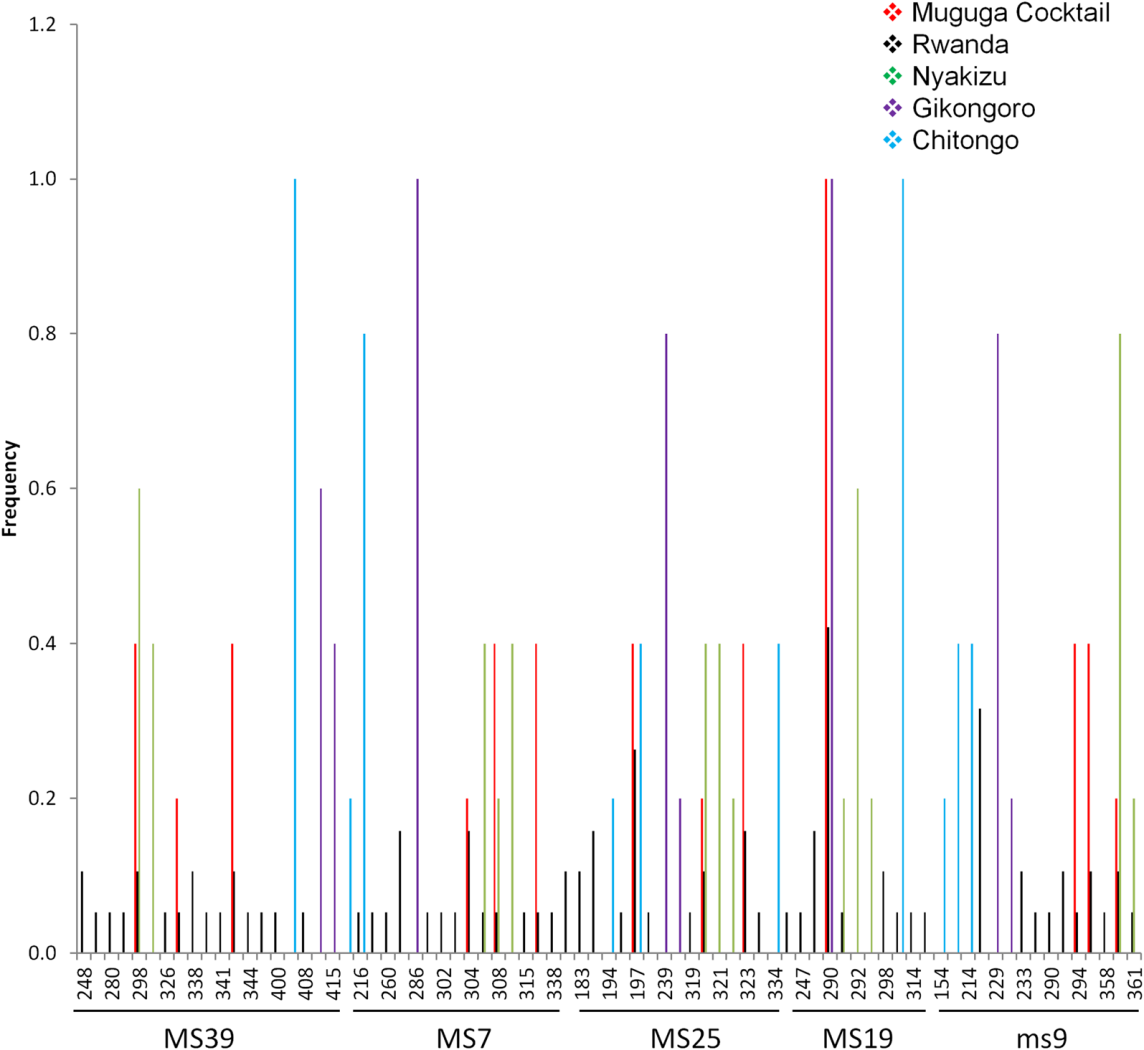
Overall allele frequency from field samples, Muguga, Kiambu and Serengeti (together referred to Muguga cocktail), Chitongo, Gikongoro and Nyakizu vaccine isolates showing 41, 10, 8 and 6 unique alleles from Rwanda field samples, Chitongo, Gikongoro and Nyakizu vaccine isolates, respectively. A higher proportion of unique alleles is observed as compared to shared alleles. Predominant alleles were calculated as proportions of the total of each satellite marker and histograms were generated from the multi-locus genotype. The numbers on the x-axis represent the allele sizes in base pairs observed on each locus

**Fig. 7:**
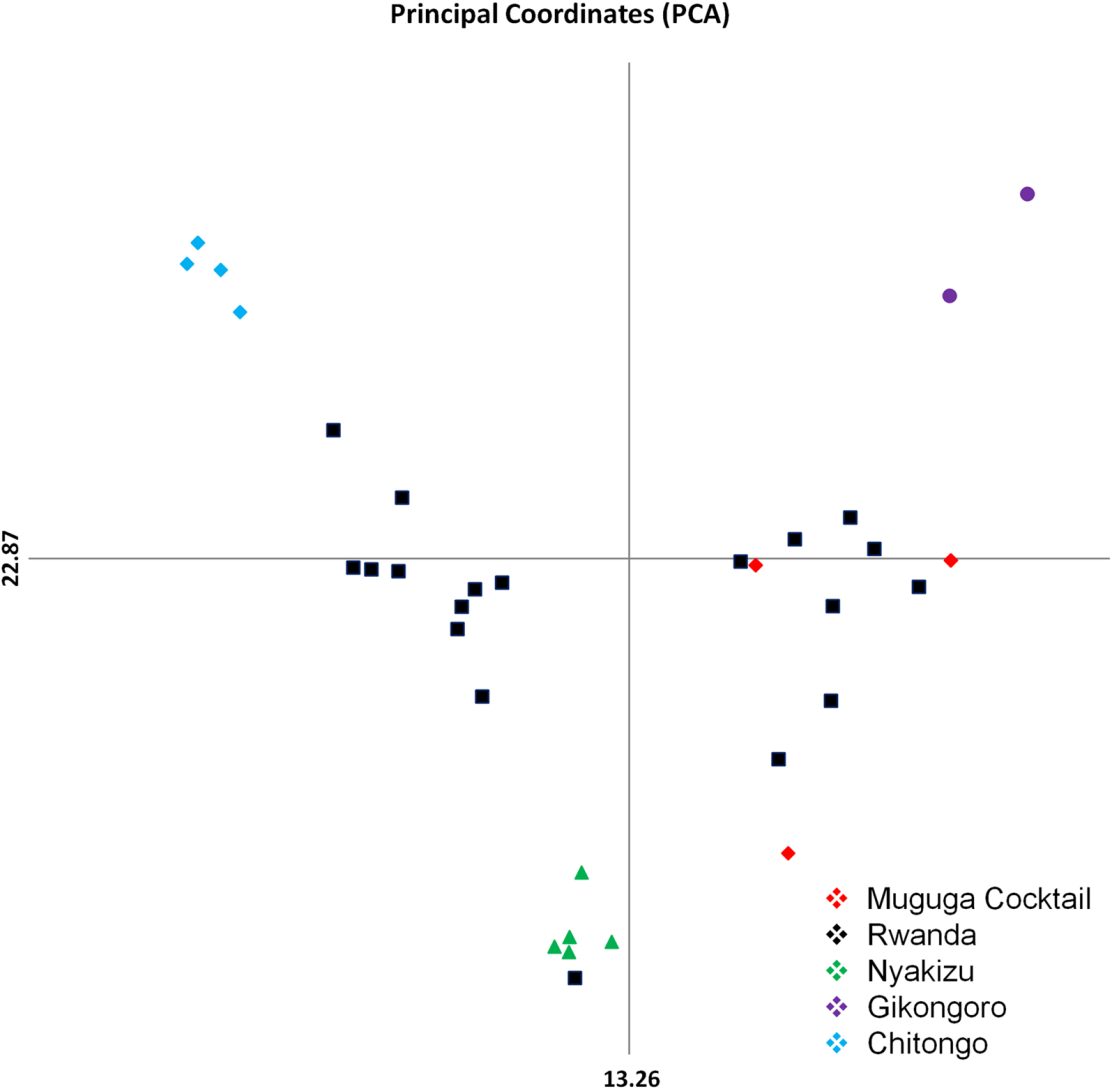
Principal components analysis (PCA) of *Theileria parva* field populations from Bugesera District, Eastern Rwanda and Muguga, Kiambu and Serengeti (collectively referred to as Muguga cocktail), Chitongo, Gikongoro and Nyakizu isolates showing a level of sub-structuring. Multilocus genotype data was used to construct the PCA and in each axis, the numbers show the variation in proportion in the population data set

A selection of 4 (MS39, MS25, MS7 and MS19) polymorphic microsatellite and one mini-satellite (ms9) markers representing the 4 chromosomes of *T. parva* were utilized to genotype 19 field samples as well as the Muguga, Kiambu, Serengeti, Chitongo, Nyakizu and Gikongoro vaccine isolates. Within the field samples, the highest number of alleles observed was 19 and this was on locus MS39 (Fig. 5a) followed by 18, 16, 15 and 13 on loci MS7, MS25, ms 9 and MS19, respectively (Fig. 5, Table 4), thus MS39 was the most polymorphic marker. The gene diversities were similar except for MS19 which produced a value of zero when Muguga, Kiambu and Serengeti were treated as one population referred to as Muguga cocktail (MC) (Table 4). For the vaccine isolates, a maximum number of 3 alleles were observed (Fig. 5, Table 4). Shared alleles between field samples and the MC or Nyakizu were also observed (Fig. 5). Between field samples and MC, 3 alleles were shared on loci MS39, MS7, MS25 and ms9 and 1 allele on locus MS19 (Fig. 5). Further, between field samples and Nyakizu, 2 alleles were shared on MS7 and ms9, and 1 allele on MS39, MS25 and MS19. Gikongoro and Chitongo isolates did not share any alleles with either the field samples or MC and Nyakizu isolates. Overall, a total of 81 alleles were observed across all loci with 16 being shared and 41, 10, 8 and 6 being unique to field samples, Chitongo, Gikongoro and Nyakizu, respectively (Fig. 6). In addition, principal components analysis (PCA) (Fig. 7) was utilized to assess the similarity of the field samples with MC, Chitongo, Nyakizu and Gikongoro isolates. On PCA, the field samples were present in all four quadrants. Some extent of independent clustering was observed in field samples with 8 occupying the right quadrants together with MC and 11 occupying the left quadrants (Fig. 7). Gikongoro isolate occupied the right upper quadrant while Chitongo and Nyakizu occupied the left upper and lower quadrants, respectively. Based on PCA, the field samples were more closely related to MC as compared to Gikongoro, Chitongo and Nyakizu isolates. Further, the field samples as well as the vaccine isolates appeared to represent slightly different populations and this coupled with the high number of unique alleles observed in Figs. 5 and 6 was indicative of genetic sub structuring. However, no further investigations were performed to determine whether these were separate populations or not due to the low number of samples under study. Nevertheless, owing to the closeness of the field samples with MC, the F_ST_ value, estimated heterozygosity, mean number of genotypes as well as the level of linkage equilibrium were assessed. The F_ST_ value calculated was 0.037 and indicated low genetic differentiation (Table 5). The estimated heterozygosity and mean number of genotypes per loci for field samples and MC were 0.906 and 8.311, and 0.640 and 2.422, respectively. The total estimated heterozygosity for the field samples and MC was 0.773 while the mean number of genotypes per loci was 5.367 (Table 5). Furthermore, linkage equilibrium (LE) between the field samples and MC was assessed by measuring the LE levels of the alleles at loci pairs using the standard index of association (ISA). A ISA value of 0.1887 (*P* < 0.01) and a V_D_ value of 0.9258, which was greater than the L value of 0.6058 were obtained indicating absence of linkage equilibrium and random mating (Table 5). Overall, microsatellite analysis of Rwanda samples and vaccine isolates indicated that the MC was the most closely related population to the field samples in Rwanda.

**Table 4:** Variation of *T. parva* alleles from Rwanda field samples, Muguga, Kiambu and Serengeti (collectively referred to as Muguga cocktail), Chitongo, Gikongoro and Nyakizu vaccine isolates

	Population	*n*	MS39	MS7	MS25	MS19	ms9
Gene diversity	Muguga cocktail	5	0.800	0.800	0.800	0.00	0.800
	Rwanda	19	0.988	0.965	0.930	0.889	0.889
	Chitongo	5	0.000	0.400	0.800	0.000	0.800
	Gikongoro	5	0.600	0.000	0.400	0.000	0.400
	Nyakizu	5	0.600	0.800	0.800	0.700	0.400
No. of alleles	Muguga cocktail	5	3	3	3	1	3
	Rwanda	19	15	14	9	9	10
	Chitongo	5	1	2	3	1	3
	Gikongoro	5	2	1	2	1	2
	Nyakizu	5	2	3	3	3	2
	Total	34	19	18	16	13	15

*Abbreviation*: n, number of samples

**Table 5:** Genetic analyses of Rwanda field samples and Muguga, Kiambu and Serengeti (collectively referred to as Muguga cocktail) vaccine isolates

Population	No. of genotypes/loci	Estimated heterozygosity	F_ST_	V_D_	L	*P*-value	ISA
Rwanda	8.311	0.906					
Muguga cocktail	2.422	0.640					
Overall	5.367	0.773	0.037	0.9258	0.6058	<0.01	0.1887

*Abbreviation:* ISA, standard index of association; V_D_, mismatch variance (linkage analysis); L, upper 95% confidence limit of Monte Carlo simulation (linkage analysis)

### Viability of the vaccine

To confirm the viability of the vaccine, two naïve animals were challenged with 1 ml of a 1:10 dilution of the MCL01 vaccine and severe reactions as measured against the ECF reaction index scores [37] were observed.

### Safety of the vaccine

Serology revealed that 35 out of 41 (85.4%) immunized cattle seroconverted and no adverse reactions were observed during the entire period of the study.

### Efficacy of Muguga cocktail stabilate as a vaccine against East Coast fever in cattle

Out of a total of 41 immunized calves, 25 were non-reactive while only 6 presented with severe reaction meaning that the fraction of severe reactors in the vaccinated group was 6/41. On the other hand, from a total of 40 non-immunized calves, 32 presented with severe reactions while only 5 were non-reactive, giving a fraction of severe reactors in the control group as 32/40 (Table 6). The vaccine efficacy was therefore calculated as 81.7% indicating effective protection in the immunized animals.

**Table 6:** Evaluation of MCL01 under field conditions in Rwanda

Group	No. of animals	NR	MR	MODR	SR
Non-immunized	40	5	2	1	32
Immunized	41	25	7	3	6

*Abbreviation:* NR, non-reactor; MR, mild reactor; MODR, moderate reactor; SR, severe reactor

## Discussion

In order to implement an effective immunization programme against *T. parva* in regions that lack such programmes and to prevent occurrences such as the ECF outbreak in the Comoros Islands [47], it is imperative to conduct studies that provide information on the strains of *T. parva* present in that particular region [28] prior to introduction of ITM. This approach is favored because *T. parva* immunization only provides strain specific protection and transmission of vaccine components to unvaccinated animals has been shown to occur [29, 48–50], consequently introducing new strains to a region and setting the platform on which recombination with local strains might occur, producing new parasite strains and thus causing disease. Furthermore, immunization against *T. parva* introduces carrier status in the immunized animals and these might also act as sources of future infection. In contrast to other studies [25, 28, 29, 51, 52], we extensively investigated the extent of sequence diversity, phylogenetic relationships and evolutionary dynamics of *Tp1* and *Tp2* genes coupled with satellite analysis using a panel of five satellite markers to determine the similarities among the field samples collected from Bugesera District of Rwanda and the Muguga, Kiambu, Serengeti, Chitongo, Gikongoro and Nyakizu vaccine isolates. The data generated from these analyses was then used to inform field challenge vaccine trials in Bugesera District, Rwanda. Our concern in this study was to establish whether the combination of Muguga, Kiambu and Serengeti isolates in form of the Muguga cocktail vaccine MCL01 or the Chitongo, Gikongoro and Nyakizu vaccine isolates can be used for cattle immunization in Rwanda using a combination of sequence analysis of *Tp1* and *Tp2* genes, micro- and mini-satellite analysis and finally, field challenge trials to validate the data obtained from molecular analysis.

Based on the sequence diversity of *Tp1*, the Muguga, Kiambu and Serengeti epitope was the most abundant in field samples (11/18), followed by Chitongo (6/18) and Gikongoro (1/18) epitopes. The Nyakizu isolate shared 100% epitope sequence homology with Muguga, Kiambu and Serengeti. Further, the nucleotide diversity (0.9%) of the field samples was low, possibly due to the highly conservative nature of *Tp1* gene. In a similar manner, sequence diversity of *Tp2* equally showed a higher representation of Muguga, Kiambu and Serengeti epitope sequences in the field samples compared to Chitongo (only represented by one sample) and none for the Gikongoro isolate. Nyakizu showed high similarity with Muguga, Kiambu and Serengeti on all epitopes of *Tp2*. A high number of epitopes from field samples that were different from Muguga, Kiambu, Serengeti, Chitongo, Nyakizu and Gikongoro were also observed and this, coupled with a high nucleotide polymorphism of 15.9%, indicates the highly diverse and polymorphic nature of the *Tp2* gene [25, 28, 29, 51, 52]. Phylogenetic analysis of *Tp1* nucleotide sequences showed three clusters of cattle derived strains (Fig. 2). Field samples clustered with Muguga, Kiambu and Serengeti in cluster A and with Chitongo and Gikongoro (accession number LC507310) in cluster B. Cluster C only comprised of field samples (Fig. 2). None of the Rwanda samples clustered with buffalo derived strains in cluster D (Fig. 2). Sequences in cluster C shared 100% epitope homology with Muguga, Kiambu and Serengeti despite forming a separate cluster from these strains (Fig. 2, Additional file 2: Figure S2). This implied that even though the field samples might represent a different strain from Muguga, Kiambu and Serengeti, the immune response elicited from the immunization with a combination of Muguga, Kiambu and Serengeti known as the Muguga cocktail will most likely offer protection against these strains especially in cattle exhibiting the major histocompatibility complex (MHC) class I gene [12, 26]. The Nyakizu isolate despite demonstrating epitope sequence homology with Muguga, Kiambu and Serengeti isolates did not share the same cluster with them or any field samples from Rwanda indicating a difference on the nucleotide level between the two strains. Similarly, phylogenetic analysis of *Tp2* gene produced three main clusters M, K and N of cattle-derived strains with the first (cluster M) comprising of Muguga, Kiambu, Serengeti, Nyakizu (accession number LC507313) and Gikongoro (accession number LC507311) isolates as well as the field samples, the second (cluster K) comprising of field samples and a reference from South Sudan and the third (cluster N) comprising of Chitongo and field samples (Fig. 3). Samples in cluster K (Fig. 3) possessed *Tp2* epitope sequences different from all the vaccine isolates (Additional file 2: Figure S2) implying that these samples might belong to a different strain from the vaccine isolates used in this study. However, sample RW5 (accession number LC507276) in cluster C (Fig. 2) and cluster K (accession number LC507295) (Fig. 3) did not cluster with any of the vaccine isolates on both phylogenetic trees while the remaining samples in K (Fig. 3) clustered with Muguga, Kiambu, Serengeti and Chitongo on *Tp1* (Fig. 2) and those in C (Fig. 2) clustered with Muguga, Kiambu, Serengeti and Chitongo on *Tp2* (Fig. 3). This clustering pattern could be as a result of recombination although the most highly probable reason could be due to the presence of mixed infection by different strains in the same animal. In contrast to a recent study [51], our study revealed the presence of three main clusters of cattle derived strains in Rwanda.

A small proportion of haplotypes from the field samples from Rwanda were similar to Muguga, Kiambu and Serengeti while the majority were not (Fig. 4a, b). In the same manner, the majority of haplotypes from the field samples were also distantly related to the Chitongo, Gikongoro and Nyakizu vaccine isolates. When *Tp1* and *Tp2* sequences were concatenated, with the exception of H2, all the other haplotypes were disconnected from H1 by median vectors implying that they did not share any common ancestry with H1. In addition, a high proportion of dS compared to dN coupled with 3 and 11 negative selection sites on *Tp1* and *Tp2*, respectively and an absence of positive selection sites on both genes was indicative of the presence of purifying selection against change in dN as well as absence of selection pressure possibly attributed to the lack of vaccination programmes against ECF and/or the absence of tick control programmes. This, together with the radiating pattern observed on the MJ network further provides substantial evidence of a freely expanding population of *T. parva* with slightly similar genotypes to Muguga, Kiambu and Serengeti vaccine isolates in Rwanda, as has been reported in other regions [25, 28, 29, 51, 52].

Eukaryotes have a broad distribution of micro- and mini-satellite regions (variable number of short tanden repeats) which are under high mutation rate and do not code for any particular protein. These regions are useful for determining genetic diversity in populations [53]. In order to improve our resolution of the genotypes and understand the genetic diversity prevailing in Rwanda, we incorporated satellite analysis using five polymorphic markers encompassing the four chromosomes of the *T. parva* genome (Table 1). When Muguga, Kiambu and Serengeti isolates were treated as one population referred to as Muguga cocktail (MC), minimum diversity was observed and the number of alleles obtained was similar to previous studies [31, 49]. Similarly, minimum diversities were also observed in Chitongo, Gikongoro and Nyakizu isolates. Rwanda field samples shared a maximum of three alleles with MC, two with Nyakizu and none with Chitongo and Gikongoro isolates across the five loci (Fig. 6). The field samples also showed a relatively high number (*n* = 41) of unique alleles (Fig. 6) as well as slight independent clustering of some field samples from MC on PCA (Fig. 7), indicating a level of genetic sub-structuring. Even though this was the case, the level of sub-structuring of field samples from the MC was low (F_ST_ = 0.037), implying that the parasite populations in the field samples were similar to MC. Apart from MC, the closest vaccine population to field samples was Nyakizu (Fig. 7). Linkage equilibrium and panmixia (random mating) was not observed between field samples and MC. This is expected since the two have not interacted in a field set up; however, the sharing of alleles further indicated that a fair proportion of the genotype in the field samples was similar to MC. Apart from MC, the most similar vaccine isolate genotype to the field samples was Nyakizu while Chitongo and Gikongoro were the least similar. Moreover, Nyakizu and Gikongoro strains are native to Rwanda and were in circulation prior to the loss of a large livestock population in 1994 that could have also caused to some extent, a loss of these strains and the cattle restocking programmes that followed could have introduced new strains that were different from the native strains. However, it is also possible that the Nyakizu and Gikongoro strains could still be in circulation at a low level as evidenced by the clustering of three field samples with accession numbers LC507299, LC507303 and LC507307, with Nyakizu (accession number LC507313) on *Tp2* gene phylogenetic tree (Fig. 3) and LC507290 with Gikongoro (accession number LC507310) on *Tp1* (Fig. 2). Owing to the fact that in most field samples, the sequence and satellite data showed that the *T. parva* population in Bugesera District, Eastern Rwanda possessed a genotype that was more closely related to MC as compared to other vaccine populations and that the Nyakizu isolate when utilized in the past for ITM produced undesirable results [13], we hypothesized that the use of a cocktail vaccine combining Muguga, Kiambu and Serengeti in field conditions in Bugesera District might produce a strong immune response and protection against *T. parva.* This hypothesis was tested in a field challenge trial at Karama farm in Bugesera District using naive calves from Nyabihu District in Northern Rwanda.

To test this hypothesis, calves were screened for the major tick-borne diseases (Table 2) prior to selection for use in the study and only naïve calves were included. The rationale behind this was to avoid other tick-borne diseases from appearing in the immunized animals and causing or contributing to mortality thus interfering with the interpretation of the final immunization results. A total of 41 calves were immunized and a further 40 set as a control group. The immunized calves were further assessed for seroconversion using ELISA prior to release in the field even though antibody response following immunization does not correlate with immunity to *T. parva* since immunity is cell-mediated [24, 54]. Regardless, it is still a general belief that there is a positive correlation between high antibody titers and protective immunity and moreover, for infectious diseases, serological tests are used frequently to determine exposure or the disease state of animals. The use of ELISA assay to monitor the antibody response to infection showed higher percent positivity (PP) values in immunized than non-immunized control groups suggesting that ELISA can still be used to measure the level of response to *T. parva* infection. The high proportion of the seroconverted cattle following immunization was within the range (85–100%) considered acceptable for a viable vaccine [55] and was similar to that observed in similar studies [40, 56–58] indicating that the vaccine was able to produce similar results even under variable epidemiological and geographic areas. Further, the Muguga cocktail vaccine administered to calves as recommended [38] had an efficacy of 81.7% and was almost similar to the 82% reported in Machakos, Kenya [59] but lower than the 95% and 97% previously reported by ILRI [60] and Babo Martins et al. [40], respectively. This could probably be attributed to differences in tick challenge between study sites, differences in the vector tick infection rates, and environmental factors. In addition, Karama experimental site has been reported to have heavy tick loads on animals [39] and coupled with high infection rates in ticks could probably explain the 14.7% ECF fatal cases recorded in the immunized group. Another possibility that requires further investigation could be that there was a presence of breakthrough strains in the exposure field. This unfortunately was not followed through due to unforeseen circumstances, nonetheless, the immunization trial results still indicate that the vaccine had a significant protective effect and reduced the incidence of ECF by 81.7%. It can also be argued that the non-reactor animals had a natural protection against ECF. The alternative scenario would suggest that these animals did not get a chance to be in contact with infected ticks. Whatever the case, vaccination of all calves/yearlings in the study area would have reduced the overall incidence of the disease by 81.7%. Thus, this study does provide evidence that the Muguga cocktail vaccine can be used to control ECF in Bugesera District of Eastern Rwanda and its use has a potential to greatly improve livestock production as has been the case in other parts of eastern and southern Africa where there has been a roll out of the vaccine [60, 61].

## Conclusions

Using the sequence diversity of *T. parva* *Tp1* and *Tp2* CTL antigens and mini- and microsatellite genotype data, the population of *T. parva* present in the local breed of cattle from Bugesera District was ascertained to possess a similar genotype to the components of Muguga cocktail (Muguga, Kiambu and Serengeti) vaccine and based on this, a field challenge trial was carried out which produced a vaccine efficacy of 81.7%. Therefore, this study demonstrated the importance of combining molecular characterization with field trial studies prior to the implementation of immunization campaigns and based on the 81.7% efficacy of vaccination achieved, the wide scale use of Muguga cocktail MCL01 vaccine in Bugesera District of Eastern Rwanda is likely to produce desirable results.

## Supplementary information

**Additional file 1: Table S1.** Description of field samples, Gikongoro and Nyakizu vaccine isolates used in this study.

**Additional file 2: Figure S1.** *Tp1* multiple sequence alignment of amino acids from field samples from Rwanda together with Muguga, Kiambu, Serengeti, Chitongo, Nyakizu and Gikongoro vaccine isolates. The polymorphic amino acids are highlighted, and the epitope region is presented in boxes. Rwanda field samples (*n* = 11) show 100% epitope homology with the Muguga, Kiambu and Serengeti isolates. Only one sample was similar to Gikongoro isolate while the rest were 100% similar to Chitongo epitope. **Figure S2.** *Tp2* multiple sequence alignment of amino acids from Rwanda field samples and the Muguga, Kiambu, Serengeti, Chitongo, Gikongoro and Nyakizu vaccine isolates. Field samples from Rwanda show similarity and variation across the 6 epitopes as compared to the vaccine stocks. Muguga, Kiambu and Serengeti epitopes compared to Chitongo, Gikongoro and Nyakizu are well represented in the Rwanda field samples. Majority of the samples exhibit epitope sequences that are different from Muguga, Kiambu and Serengeti sequences and only sample RW6 exhibited 100% epitope sequence homology with Chitongo vaccine isolate. The epitope region is presented in boxes and the polymorphic amino acids are highlighted.

## Data Availability

The sequences emanating from this study were deposited in the GenBank database under the accession numbers LC507273-LC507313. Additional datasets used and/or analyzed during the present study are available from the corresponding author on reasonable request.

## Abbreviations

CTL: cytotoxic T Lymphocytes
CTTBD: Centre for Ticks and Tick-Borne Diseases
ECF: East Coast fever
LD: linkage disequilibrium
LE: linkage equilibrium
MC: Muguga cocktail
MLG: multi-locus genotype
PCA: principal components analysis
PCR: polymerase chain reaction
RAB: Rwanda Agriculture Board
