# Supplementary material for: Sequence diversity of cytotoxic T cell antigens and satellite marker analysis of Theileria parva informs the immunization against East Coast fever in Rwanda
Source: Parasit Vectors. 2020 Sep 7;13:452. doi: 10.1186/s13071-020-04322-9 (PMC7487574; doi:10.1186/s13071-020-04322-9)
Supplement: Supplementary file 1 — Additional file 1: Table S1. Description of field samples, Gikongoro and Nyakizu vaccine isolates used in this study. [file 13071_2020_4322_MOESM1_ESM.docx]

**Additional file 1: Table S1: Description of field samples, Gikongoro and Nyakizu vaccine isolates used in this study**

| Sample | Year of collection | Host | Origin | Tp1 Accession number | Tp2 Accession number |
| --- | --- | --- | --- | --- | --- |
| RW1 | 2015 | Cattle | Bugesera District | LC507273 | LC507291 |
| RW2 | 2015 | Cattle | Bugesera District |  | LC507292 |
| RW3 | 2015 | Cattle | Bugesera District | LC507274 | LC507293 |
| RW4 | 2015 | Cattle | Bugesera District | LC507275 | LC507294 |
| RW5 | 2015 | Cattle | Bugesera District | LC507276 | LC507295 |
| RW6 | 2015 | Cattle | Bugesera District | LC507277 | LC507296 |
| RW7 | 2015 | Cattle | Bugesera District | LC507278 | LC507297 |
| RW8 | 2015 | Cattle | Bugesera District | LC507279 | LC507298 |
| RW9 | 2015 | Cattle | Bugesera District | LC507280 | LC507299 |
| RW10 | 2015 | Cattle | Bugesera District | LC507281 | LC507300 |
| RW11 | 2015 | Cattle | Bugesera District | LC507282 | LC507301 |
| RW12 | 2015 | Cattle | Bugesera District | LC507283 | LC507302 |
| RW13 | 2015 | Cattle | Bugesera District | LC507284 | LC507303 |
| RW14 | 2015 | Cattle | Bugesera District | LC507285 | LC507304 |
| RW15 | 2015 | Cattle | Bugesera District | LC507286 | LC507305 |
| RW16 | 2015 | Cattle | Bugesera District | LC507287 | LC507306 |
| RW17 | 2015 | Cattle | Bugesera District | LC507288 | LC507307 |
| RW18 | 2015 | Cattle | Bugesera District | LC507289 | LC507308 |
| RW19 | 2015 | Cattle | Bugesera District | LC507290 | LC507309 |
| Gikongoro | 2003 | Cattle | Nyamagabe District | LC507310 | LC507311 |
| Nyakizu | 2003 | Cattle | Nyaruguru District | LC507312 | LC507313 |
