# Supplementary material for: Sequence diversity of cytotoxic T cell antigens and satellite marker analysis of Theileria parva informs the immunization against East Coast fever in Rwanda
Source: Parasit Vectors. 2020 Sep 7;13:452. doi: 10.1186/s13071-020-04322-9 (PMC7487574; doi:10.1186/s13071-020-04322-9)

Additional file 2: Figure S1

```
JF451938. Muguga     1 MATSIAFAADPGFCYFLLIPGPDSKPIFFKNDGDKFLRCVGYPKVKEEMIEMATKFNRLPKGVEIPAPPGVKPEAPTPTPTTITPSVPPT 90
JF451939. Kiambu     1 MATSIAFAADPGFCYFLLIPGPDSKPIFFKNDGDKFLRCVGYPKVKEEMIEMATKFNRLPKGVEIPAPPGVKPEAPTPTPTTITPSVPPT 90
JF451940. Serengeti  1 MATSIAFAADPGFCYFLLIPGPDSKPIFFKNDGDKFLRCVGYPKVKEEMIEMATKFNRLPKGVEIPAPPGVKPEAPTPTPTTITPSVPPT 90
Nyakizu B66          1 MATSIAFAADPGFCYFLLIPGPDSKPIFFKNDGDKFLRCVGYPKVKEEMIEMATKFNRLPKGVEIPAPPGVKPEAPTPTPTTITPSVPPT 90
RW1                  1 MATSIAFAADPGFCYFLLIPGPDSKPIFFKNDGDKFLRCVGYPKVKEEMIEMATKFNRLPKGVEIPAPPGVKPEAPTPTPTTITPSVPPT 90
RW3                  1 MATSIAFAADPGFCYFLLIPGPDSKPIFFKNDGDKFLRCVGYPKVKEEMIEMATKFNRLPKGVEIPAPPGVKPEAPTPTPTTITPSVPPT 90
RW4                  1 MATSIAFAGDPGFCYFLLIPGPDSKPIFFKNDGDKFLRCVGYPKVKEEMIEMATKFNRLPKGVEIPAPPGVKPEAPTPTPTTITPSVPPT 90
RW5                  1 MATSIAFAGDPGFWYFLLIPGPDSKPIFFKNDGDKFLRCVGYPKVKEEMIEMATKFNRLPKGVEIPAPPGVKPEAPTPTPTTITPSVPPT 90
RW6                  1 MATSIAFAADPGFCYFLLIPGPDSKPIFFKNDGDKFLRCVGYPKVKEEMIEMATKFNRLPKGVEIPAPPGVKPEAPTPTPTTITPSVPPT 90
RW7                  1 MATSIAFARDPGFCYFLLIPGPDSKPIFFKNDGDKFLRCVGYPKVKEEMIEMATKFNRLPKGVEIPAPPGVKPEAPTPTPTTITPSVPPT 90
RW8                  1 MATSIAFAADPGFCYFLLIPGPDSKPIFFKNDGDKFLRCVGYPKVKEEMIEMATKFNRLPKGVEIPAPPGVKPEAPTPTPTTITPSVPPT 90
RW9                  1 MATSIAFAGDPGFCYFILIPGPDSKPIFFKNDGDKFLRCVGYPKVKEEMIEMATKFNRLPKGVEIPAPPGVKPEAPTPTPTTITPSVPPT 90
RW13                 1 MATSIAFAGDPGICYFLLIPGPDSKPIFFKNDGDKFLRCVGYPKVKEEMIEMATKFNRLPKGVEIPAPPGVKPEAPTPTPTTITPSVPPT 90
RW14                 1 MATSIAFAADPGFCYFLLIPGPDSKPIFFKNDGDKFLRCVGYPKVKEEMIEMATKFNRLPKGVEIPAPPGVKPEAPTPTPTTITPSVPPT 90
RW17                 1 MATSIAFAADPGFCYFLLIPGPDSKPIFFKNDGDKFLRCVGYPKVKEEMIEMATKFNRLPKGVEIPAPPGVKPEAPTPTPTTITPSVPPT 90
Gikongoro RW1        1 MATSIAFAADPGFCYFLLIPGPDSKPIFFKNDGDKFLRCVGYPKVKEEMIEMATKFNRLPKGVEIPAPPGVKPEAPTPTPTTITPSVPPT 90
RW19                 1 MATSIAFAADPGFCYFLLIPGPDSKPIFFKNDGDKFLRCVGYPKVKEEMIEMATKFNRLPKGVEIPAPPGVKPEAPTPTPTTITPSVPPT 90
RW10                 1 MATSIAFAGDPGFCYFLIIPGPDSKPIFFKNDGDKFLRCVGYPKVKEEIIEMATKFNRLPKGVEIPAPPGVKPEAPTPTPTTITPSVPPT 90
RW11                 1 MATSIAFAADPGFCYFLLIPGPDSKPIFFKNDGDKFLRCVGYPKVKEEIIEMATKFNRLPKGVEIPAPPGVKPEAPTPTPTTITPSVPPT 90
JF451975 Chitongo Z2 1 MATSIAFAADPGFCYFLLIPGPDSKPIFFKNDGDKFLRCVGYPKVKEEIIEMATKFNRLPKGVEIPAPPGVKPEAPTPTPTTITPSVPPT 90
RW12                 1 MATSIAFAGDPGFCYFILIPGPDSKPIFFKNDGDKFLRCVGYPKVKEEIIEMATKFNRLPKGVEIPAPPGVKPEAPTPTPTTITPSVPPT 90
RW15                 1 MATSIAFAGDPGFCYFLLIPGPDSKPIFFKNDGDKFLRCVGYPKVKEEIIEMATKFNRLPKGVEIPAPPGVKPEAPTPTPTTITPSVPPT 90
RW16                 1 MATSIAFAGDPGFCYFLLIPGPDSKPIFFKNDGDKFLRCVGYPKVKEEIIEMATKFNRLPKGVEIPAPPGVKPEAPTPTPTTITPSVPPT 90
RW18                 1 MATSIAFAADPGFCYFLLIPGPDSKPIFFKNDGDKFLRCVGYPKVKEEIIEMATKFNRLPKGVEIPAPPGVKPEAPTPTPTTITPSVPPT 90
```

Additional file 2: Figure S2

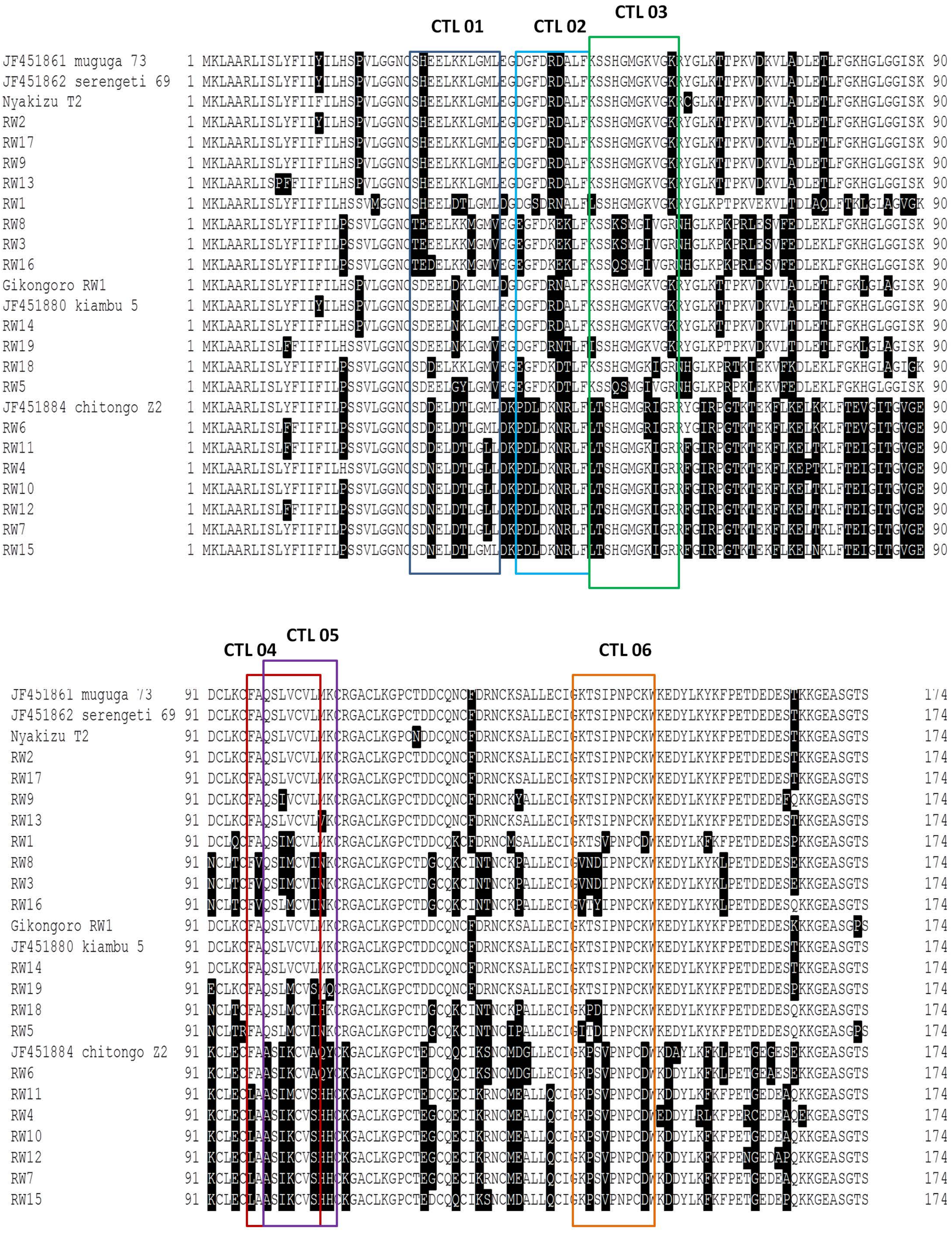

Supplement: Supplementary file 2 — Additional file 2: Figure S1. Tp1 multiple sequence alignment of amino acids from field samples from Rwanda together with Muguga, Kiambu, Serengeti, Chitongo, Nyakizu and Gikongoro vaccine isolates. The polymorphic amino acids are highlighted, and the epitope region is presented in boxes. Rwanda field samples (n = 11) show 100% epitope homology with the Muguga, Kiambu and Serengeti isolates. Only one sample was similar to Gikongoro isolate while the rest were 100% similar to Chitongo epitope. Figure S2. Tp2 multiple sequence alignment of amino acids from Rwanda field samples and the Muguga, Kiambu, Serengeti, Chitongo, Gikongoro and Nyakizu vaccine isolates. Field samples from Rwanda show similarity and variation across the 6 epitopes as compared to the vaccine stocks. Muguga, Kiambu and Serengeti epitopes compared to Chitongo, Gikongoro and Nyakizu are well represented in the Rwanda field samples. Majority of the samples exhibit epitope sequences that are different from Muguga, Kiambu and Serengeti sequences and only sample RW6 exhibited 100% epitope sequence homology with Chitongo vaccine isolate. The epitope region is presented in boxes and the polymorphic amino acids are highlighted. [file 13071_2020_4322_MOESM2_ESM.pdf]
